# Case Report: Spontaneous complete uterine rupture in the second and third trimester of pregnancy

**DOI:** 10.3389/fmed.2026.1802146

**Published:** 2026-04-15

**Authors:** Yanlin Mou, Huan Yang, Qin Zhong

**Affiliations:** 1 Reproductive Medicine Center, Department of Obstetrics and Gynecology, The Third People’s Hospital of Chengdu, Chengdu, Sichuan, China; 2 Mingnuo Medical Comprehensive Outpatient Department of Chengdu, Chengdu, Sichuan, China

**Keywords:** non-labor rupture, non-scar uterine, placenta accreta spectrum, spontaneous complete uterine rupture, the second and third trimester of pregnancy

## Abstract

**Objective:**

To summarize the clinical features, risk factors, and maternal and fetal outcomes of spontaneous complete uterine rupture during the second and third trimester of pregnancy, and to explore key aspects of early identification and management, especially in cases without prior cesarean section. This study aims to provide evidence-based insights for early warning and emergency intervention in clinical practice.

**Methods:**

This is a retrospective case series analysis of seven cases of spontaneous complete uterine rupture occurring in the second and third trimester of pregnancy. We analyzed patient demographics, obstetric history, gestational age at rupture, clinical presentation, management strategies, and outcomes. Descriptive statistical methods were employed, with continuous variables expressed as medians (range) and categorical variables as frequencies (percentages).

**Results:**

The median age of the seven women was 30 years old, with a median gestational age at rupture of 27 weeks. Among them, 57.1% (4/7) experienced rupture in the second trimester (<28 weeks), and 71.4% (5/7) had non-labor ruptures. 42.9% (3/7) of the cases involved women without a previous cesarean section. The most common clinical symptom was sudden onset of abdominal pain, often accompanied by shoulder pain, abnormal fetal heart rate, or vaginal bleeding, though these symptoms were non-specific. Surgical confirmation revealed rupture sites in the lower uterine segment (3 cases), fundus (3 cases), and cornua (1 case). Hemorrhagic shock (blood loss ≥ 2000 mL) occurred in 85.7% (6/7) of cases, with three cases complicated by placenta accreta spectrum (PAS). While all mothers survived, the perinatal mortality rate was 85.7% (6/7), with only one surviving fetus.

**Conclusion:**

Spontaneous complete uterine rupture during the second and third trimester of pregnancy typically presents as non-labor acute abdominal pain and may occur in women without a prior uterine surgery history, especially in the second trimester, where fetal outcomes are poor. Clinicians should maintain a high index of suspicion for uterine rupture in pregnant women presenting with acute abdominal pain, regardless of previous cesarean history. Early diagnosis and the establishment of a rapid, multidisciplinary emergency response are critical to improving maternal and fetal outcomes.

## 1 Introduction

Spontaneous complete uterine rupture refers to the full-thickness rupture of the uterine wall (including the endometrium, myometrium, and serosa) without external trauma or iatrogenic injury, leading to communication between the uterine cavity and the abdominal cavity. This is a life-threatening obstetric emergency for both mother and fetus (1). The most common high-risk scenario is a trial of labor after cesarean (TOLAC) delivery, with the incidence of symptomatic uterine rupture ranging from 0.5% to 0.9% (2).

However, spontaneous complete uterine rupture occurring in the non-labor state is extremely rare. A large population-based study found an overall incidence of 1.6 per 10,000 pregnancies. Notably, approximately 9% of these ruptures occur in the second trimester, and a significant proportion of cases occur in women with no history of uterine surgery (3). Due to its rarity, most existing literature on this condition consists of case reports, and there is a lack of systematic summaries and analyses regarding the clinical features, warning signs, and maternal-fetal outcomes of spontaneous complete uterine rupture during the second and third trimester of pregnancy, particularly in cases involving women without prior uterine surgery. This knowledge gap may lead to delayed recognition and passive management in clinical practice, resulting in catastrophic outcomes.

Therefore, this study aims to summarize the clinical data of seven cases of spontaneous complete uterine rupture during the second and third trimester of pregnancy treated at our institution through a retrospective case series analysis. The study focuses on high-risk factors, clinical presentations (especially non-labor and non-specific symptoms), rupture site locations, and maternal-fetal outcomes. By revealing the true characteristics of this rare obstetric emergency, we hope to provide valuable experience for enhancing early warning and multidisciplinary emergency response capabilities, ultimately improving perinatal outcomes.

## 2 Materials and methods

### 2.1 Study design

This study is a single-center, retrospective case series analysis.

### 2.2 Case definition and selection

**FIGURE 1:**
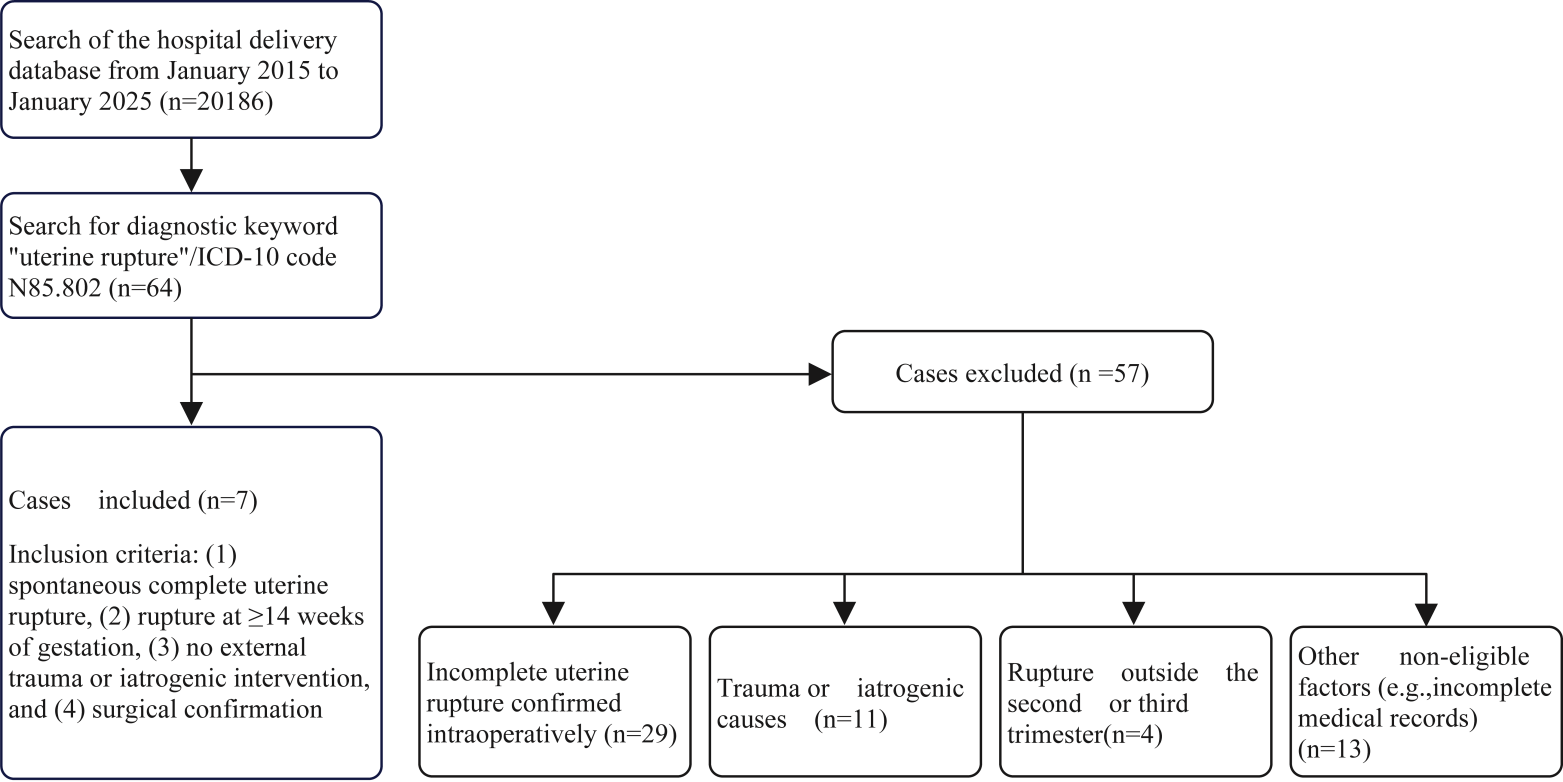
Case identification and selection process. Flowchart illustrating the steps taken to identify and select cases related to uterine rupture diagnoses. The process begins with a hospital database search, followed by case screening, exclusion based on specific criteria, and final case inclusion (*n* = 7).

We retrospectively analyzed all cases of spontaneous complete uterine rupture during the second and third trimester of pregnancy treated at our institution from January 2015 to January 2025. Spontaneous complete uterine rupture was defined as full-thickness rupture of the uterine wall (endometrium, myometrium, and serosa) occurring at a gestational age ≥ 14 weeks, without any clear history of external trauma or iatrogenic intervention, and confirmed by surgical exploration. Case selection was performed by searching the hospital’s electronic medical records (EMR) system using the diagnostic keyword “uterine rupture” and ICD-10 code (N85.802) for the period from January 2015 to January 2025. A total of 64 records were retrieved and reviewed based on the inclusion and exclusion criteria. 7 cases met the inclusion criteria and were included in the analysis. The case selection process and reasons for exclusions are shown in the flow diagram (Figure 1).

### 2.3 Data collection

Data were independently extracted from the hospital’s electronic medical records system by two researchers. The following variables were collected: demographic characteristics, detailed obstetric history, gestational age at rupture and clinical presentation, diagnostic and treatment process (including surgical records), intraoperative findings (rupture site and blood loss), complications, and maternal and fetal outcomes. All data were cross-checked to ensure accuracy.

### 2.4 Statistical analysis

Descriptive statistical methods were used. Continuous variables are expressed as median (range), and categorical variables are expressed as frequency (percentage).

## 3 Results

### 3.1 Patient baseline characteristics and risk factors

A total of seven cases of spontaneous complete uterine rupture during the second and third trimester of pregnancy were included in this study. The median age of the patients was 30 years old (range: 27–39 years old), and the median gestational age at rupture was 27 weeks (range: 20–39 weeks). Notably, more than half (4/7, 57.1%) of the ruptures occurred in the second trimester (<28 weeks). Of these, 4 patients (57.1%) had a history of cesarean section, with 3 cases occurring in the second trimester. The remaining 3 patients had no history of uterine surgery, and all had at least one previous vaginal delivery. Detailed baseline characteristics are shown in Table 1.

**TABLE 1:** Clinical, surgical, and perinatal data of uterine rupture cases.

Serial No.	Age (years)	Gravidity/parity	Gestational age at rupture (weeks)	Interval since last cesarean section (years)	Regular antenatal care (yes/no)	Emergency transfer from referring hospital (yes/No)	Labor onset (yes/no)	Clinical manifes-tations and exami-nations	Surgical findings	Estimated blood loss (mL)	Blood trans-fusion (Yes/No)	Surgical procedure	Surgical compli-cations	Past history and pregnancy compli-cations	Perinatal outcomes
1	39	4 previous vaginal deliveries	38	—	Yes	Yes	Yes	Abdominal pain and vaginal discharge for several days, worsening for >6 h, fetal descent stalled in second stage of labor	15 cm horizontal rupture in lower anterior uterine segment, fetus expelled into abdominal cavity with fetal head in vaginal canal	2000	Yes	Total hysterectomy	Urinary retention, Hemorrhagic shock	No significant	Maternal survival was achieved, but fetal death occurred
2	30	1 previous cesarean section	27	10	No	Yes	No	Recurrent abdominal pain for >8 h, epigastric pain, referred shoulder pain, dyspnea, ultrasound showed massive free fluid	Gourd-shaped uterus, placenta percreta involving anterior uterine wall and fundus with hemorrhage	3000	Yes	Subtotal hysterectomy	Hemorrhagic shock	No significant	Maternal survival was achieved, but fetal death occurred
3	27	2 previous cesarean sections, 1 induced abortion	25	3	Yes	Yes	No	Abdominal pain with diarrhea for >4 h	1 cm rupture in lower anterior uterine wall, placenta previa with increta	2000	Yes	Subtotal hysterectomy	Hemorrhagic shock	No significant	Maternal survival was achieved, but fetal death occurred
4	28	1 previous cesarean section	20	4	No	Yes	No	Reduced fetal movement for 1 day, lower abdominal pain and vaginal bleeding for >4 h, ultrasound-guided aspiration revealed blood-tinged fluid	1 cm rupture at uterine scar, umbilical cord and fetal shoulder visible	400	No	Uterine repair	No significant	No significant	Maternal survival was achieved, but fetal death occurred
5	33	2 previous vaginal deliveries	39	—	No	Yes	Yes	Early labor progressed smoothly, sudden drop in fetal heart rate to 70 bpm, suggestive of asynclitic presentation and fetal distress	4 cm rupture in right anterior uterine wall, protruding placental tissue, hematoma	3000	Yes	Total hysterectomy	Hemorrhagic shock	No significant	Maternal survival was achieved, but fetal death occurred
6	35	1 previous vaginal delivery	27	—	Yes	Yes	No	Abdominal pain for >12 h, progressively worsening, radiating to right shoulder	20 cm × 16 cm × 3 cm pelvic blood clot with intra-abdominal hemorrhage, 12 cm rupture of uterine fundus with placenta accreta	5200	Yes	Uterine repair	Hemorrhagic shock, Multiple organ dysfunction	Gestational diabetes mellitus	Maternal survival was achieved, but fetal death occurred
7	30	1 previous cesarean section, 1 induced abortion	35	2	Yes	Yes	No	Persistent lower abdominal pain for >10 h	4 cm rupture at right lateral uterine fundus near cornu	2800	Yes	Uterine repair	Hemorrhagic shock	History of laparoscopy for infertility and appendectomy, Antiphospholipid syndrome, undifferentiated connective tissue disease, subclinical hypothyroidism, and autoimmune hepatitis diagnosed during pregnancy	The mother was successfully resuscitated, and a live birth was achieved. The newborn’s Apgar scores were 2-7- 8

Estimated blood loss was assessed using visual estimation, gravimetric, and volumetric methods. Hemorrhagic shock was defined as blood loss > 1500 mL (approximately 30%–40% of total blood volume) or the presence of hemodynamic instability requiring urgent resuscitation. Timing from symptom onset to surgery was unavailable for emergency referral cases.

**Serial No.**: Patient identifier. **Age (years)**: Patient’s age. **Gravidity/ Parity**: Total number of pregnancies and live births. **Gestational Age at rupture (weeks)**: Gestational age at the time of rupture. **Interval Since Last Cesarean Section (years)**: Time since the last Cesarean section. **Regular Antenatal Care (Yes/No)**: Whether the patient received regular antenatal care. **Emergency Transfer From Referring Hospital (Yes/No)**: Whether the patient was transferred as an emergency from a referring hospital. **Labor onset (Yes/No)**: Whether labor started before rupture. **Clinical manifestations and examinations**: Symptoms and diagnostic findings. **Surgical Findings**: Description of rupture and surgical observations. **Estimated Blood Loss (mL)**: Estimated blood loss during surgery (in milliliters). **Blood Transfusion (Yes/No)**: Whether the patient received a blood transfusion. **Surgical Procedure**: Type of surgery performed. **Surgical Complications**: Complications during surgery. **Past History and Pregnancy Complications**: Patient’s medical and pregnancy history. **Perinatal Outcomes**: Maternal and neonatal outcomes.

### 3.2 Clinical presentation and diagnosis

In 71.4% (5/7) of the cases, uterine rupture occurred in the non-labor state. All patients presented acutely, with the most common symptom being sudden onset of abdominal pain (7/7, 100%), often accompanied by shoulder pain, abnormal fetal heart rate, or vaginal bleeding. However, the symptom combinations were varied and non-specific. Only 2 cases (28.6%) of uterine rupture in women with previous cesarean section were suspected preoperatively based on clinical presentation and ultrasound findings (suggesting intra-abdominal fluid and interruption of myometrial continuity). The remaining 5 cases (71.4%) were diagnosed during exploratory laparotomy performed for suspected other acute abdominal or obstetric emergencies. The median time from symptom onset to surgery was 6 h (range: 1–12 h).

### 3.3 Surgical findings and maternal-fetal outcomes

Surgical exploration confirmed complete uterine rupture in all cases. The rupture sites were located in the lower uterine segment (3 cases), fundus (3 cases), and cornu (1 case). All patients experienced severe postpartum hemorrhage, with 6 cases (85.7%) progressing to hemorrhagic shock (blood loss ≥ 2000 mL), and all required blood transfusions. Two cases (28.6%) had placenta accreta. To control bleeding, 4 patients underwent hysterectomy (2 subtotal hysterectomies and 2 total hysterectomies), while the remaining 3 cases underwent uterine repair. Despite aggressive resuscitation, the perinatal mortality rate was extremely high, at 85.7% (6/7), with only one surviving neonate in a late-term pregnancy. All mothers survived after multidisciplinary resuscitation, with no maternal deaths. Detailed clinical processes, management, and outcomes are shown in Table 1. A timeline of clinical progression, highlighting key events and interventions, is provided in Table 2.

**TABLE 2:** Timeline of clinical progression.

Serial no.	Symptom onset	Transfer (yes/no)	Rupture diagnosis	Surgical intervention	Outcomes
1	Pain + labor arrest	Yes	Intraoperative findings	Total hysterectomy	Maternal survive / Fetal death
2	Pain + shock	Yes	Ultrasound	Subtotal hysterectomy	Maternal survive / Fetal death
3	Pain + diarrhea	Yes	Intraoperative findings	Subtotal hysterectomy	Maternal survive / Fetal death
4	Fetal movement reduce + bleeding	Yes	Ultrasound	Uterine repair	Maternal survive / Fetal death
5	Fetal heart rate drop	Yes	Intraoperative findings	Total hysterectomy	Maternal survive / Fetal death
6	Pain + shock	Yes	Intraoperative findings	Uterine repair	Maternal survive / Fetal death
7	Pain	Yes	Intraoperative findings	Uterine repair	Maternal survive / Live birth

**Serial No.**: Patient identifier. **Symptom Onset:** Initial symptoms observed, including pain, labor arrest, shock, etc. **Transfer (Yes/No):** Whether the patient was transferred as an emergency from a referring hospital. **Rupture Diagnosis:** Method used for diagnosing uterine rupture, including intraoperative findings or ultrasound (US). **Surgical Intervention:** Type of surgery performed, such as total hysterectomy, subtotal hysterectomy, or uterine repair. **Outcomes:** Maternal and fetal outcomes, indicating whether the mother survived and the status of the fetus (death or live birth).

## 4 Discussion

### 4.1 Incidence and epidemiology of uterine rupture

Uterine rupture is a rare but potentially life-threatening obstetric complication. Population-based registry studies have shown that the incidence of uterine rupture varies based on uterine scar status and obstetric management strategies. Data from the Norwegian Medical Birth Registry, which includes over 1.3 million deliveries, reported an incidence of complete uterine rupture of approximately 0.38 per 10,000 deliveries in women without a previous cesarean section, whereas the incidence increased to 21.1 per 10,000 deliveries in women with a prior cesarean scar (3). Similarly, a cohort study using the Danish Medical Birth Registry reported an incidence of 3.3 per 100,000 deliveries in women without a prior cesarean section (4). Additionally, recent multinational registry data from European obstetric surveillance systems further demonstrate that non-labor uterine rupture is extremely rare in women with an unscarred uterus, with an estimated incidence of approximately 0.03 per 10,000 pregnancies (5).

In comparison to these population-based studies, our case series focuses on spontaneous uterine rupture occurring during the second and third trimesters of pregnancy. In our study, 3 cases occurred in women with a prior cesarean scar, and 4 cases occurred in women without a cesarean history. While population-based studies report a low incidence of non-labor uterine rupture, our study found that 5 out of 7 cases occurred in a non-labor state, highlighting the diagnostic challenges associated with non-labor uterine rupture during the second and third trimesters. These findings offer valuable insights for clinicians, emphasizing the need to consider diverse clinical pathways in the diagnosis and management of such rare cases, ultimately improving the possibility of early recognition and intervention.

### 4.2 Second trimester and non-labor rupture

Traditional obstetric focus has predominantly been on uterine rupture during labor at term, especially in the context of trial of labor after cesarean (TOLAC), which aims to reduce cesarean section rates and related complications (6). This narrow focus may result in the underappreciation of earlier and subtler forms of rupture, which can easily be overlooked. Spontaneous uterine rupture during the second and early third trimesters, especially in the non-labor state, is rare but life-threatening. However, comprehensive epidemiological data on the incidence and risk factors for such ruptures is lacking. In this study, the median gestational age at rupture was 27 weeks, with 57.1% (4/7) of ruptures occurring in the second trimester (<28 weeks), and 71.4% (5/7) occurring in the non-labor state. This highlights the critical, yet often underestimated, nature of the second trimester as a high-risk period. Though non-labor uterine rupture is uncommon, it is associated with significantly higher risks of uterine removal and perinatal death, with the second trimester being a particularly dangerous window for these outcomes (7).

Uterine rupture can present with a wide range of symptoms, depending on factors such as the underlying cause, rupture site, gestational age, and blood loss. Common clinical manifestations include abdominal pain, vaginal bleeding, and abnormal fetal heart rate patterns (8). In some cases, rupture occurs without clear warning signs and is identified incidentally during routine prenatal care or cesarean section, with findings such as myometrial disruption or membrane prolapse (9). During labor, uterine rupture typically presents with features like “pathological retraction ring” and abnormal fetal monitoring, whereas non-labor rupture is rarer and usually presents with insidious, nonspecific symptoms (10). These nonspecific symptoms can lead to diagnostic delays and suboptimal management, which increases the risk of severe maternal complications (85.7% of patients in this study developed hemorrhagic shock) and high fetal mortality, especially in the second trimester (11–13).

It is particularly concerning that the fetus in the second trimester lacks the ability to survive outside the uterus, and uterine rupture at this stage almost invariably results in pregnancy termination. In contrast, uterine rupture in the third trimester presents a different challenge, requiring a “dual rescue” approach for both the mother and fetus, with a very narrow therapeutic window. Clinicians must maintain a proactive mindset, with a focus on early recognition of risk factors and prompt, gestational age-specific intervention.

Recent evidence indicates that uterine rupture may present with atypical or subclinical manifestations, complicating timely diagnosis. Matteo Terrinoni et al. (14) reported an incidental uterine scar dehiscence during an elective cesarean section in a high-risk pregnancy, without significant abdominal pain, hemorrhage, or cardiotocographic abnormalitiesa. This suggests that rupture may manifest with subtle or insidious symptoms rather than the classic triad of acute abdominal pain, fetal distress, and vaginal bleeding. In patients presenting with acute abdominal pain during the second and third trimester, differential diagnoses include placental abruption, acute surgical abdomen (e.g., appendicitis), uterine torsion, and other obstetric emergencies. Diagnostic challenges arise from overlapping clinical features and the critical condition of several externally referred patients. Diagnosis relied on integrating clinical presentation, rapid clinical deterioration, available imaging findings, and intraoperative confirmation. In several cases, definitive diagnosis was established only during surgery, highlighting the importance of maintaining a high index of suspicion and being prepared for prompt surgical intervention in high-risk pregnancies.

### 4.3 Non-scar uterine rupture

Scarred uteri are widely recognized as the primary risk factor for uterine rupture due to the thinner and less elastic tissue at the scar site compared to normal myometrium. As the uterus expands in subsequent pregnancies, the scar tissue is further stretched, increasing the risk of rupture and. In this study, 57.1% (4/7) of cases involved scarred uteri, which aligns with previous literature findings (15–17).

Non-scar uterine rupture, traditionally considered more common in the third trimester or during labor, is often linked to obstructed labor, multiparity, and uterine structural abnormalities (18–20). However, without a clear “high-risk label,” non-scar uterine rupture is more insidious and often misdiagnosed. Unlike scarred uteri, non-scar ruptures vary greatly in terms of causative factors, rupture sites, and severity, making them harder to recognize and often leading to delayed intervention. Clinicians should remain highly vigilant for signs such as “sudden tearing abdominal pain,” “obstructed descent of the presenting part,” “fetal heart rate loss,” “gross hematuria,” and “loss of uterine contour.” If ultrasound reveals signs like “interruption of myometrial continuity,” “fetal parts in the abdominal cavity,” or “abdominal fluid,” immediate exploratory laparotomy is recommended.

The standardization of perinatal care, ultrasound protocols, and labor induction techniques is essential in reducing the incidence of uterine rupture (20). Additionally, combining clinical presentation, physical examination, and auxiliary imaging techniques can enhance the early diagnostic accuracy. When ultrasound is insufficient, MRI may serve as an additional diagnostic tool, especially in cases with anatomical changes or when ultrasound is limited (21, 22). In addition to well-recognized risk factors, we summarize each patient’s potential risk factors briefly in Table 3.

**TABLE 3:** Summary of risk factors.

Serial no.	Parity	Interpregnancy interval (years)	Prior uterine procedures	Placental location and abnormalities	Pregnancy complications
1	4	4	None	Anterior uterine segment	None
2	1	10	Cesarean section	Anterior wall, fundus, Placenta percreta	None
3	2	3	Cesarean sections, induced abortion	Lower anterior uterine wall, Placenta previa, placenta increta	None
4	1	4	Cesarean section	Posterior uterine wall	None
5	2	3	None	Right anterior uterine wall	None
6	1	5	None	Uterine fundus, Placenta accreta	Gestational diabetes mellitus
7	1	2	Cesarean section, induced abortion	Posterior uterine wall	Antiphospholipid syndrome, undifferentiated connective tissue disease, subclinical hypothyroidism, autoimmune hepatitis

**Serial No.**: Patient identifier. **Parity**: Number of live births. **Interpregnancy Interval (years)**: Time interval between the previous pregnancy and the current pregnancy (in years). **Prior Uterine Procedures**: Previous uterine surgeries or procedures, including Cesarean section, induced abortion, etc. **Placental Location and Abnormalities**: Placental location and any abnormalities, such as placenta percreta, placenta accreta, etc. **Pregnancy Complications**: Any pregnancy-related complications, including gestational diabetes, antiphospholipid syndrome, etc.

In this study, 42.9% (3/7) of uterine ruptures occurred in patients with non-scarred uteri, all of whom were multiparous. Two of these ruptures occurred at term and were associated with signs of obstructed labor, consistent with the classic link between obstructed labor and uterine rupture. However, the remaining case occurred at 29 weeks of gestation in the non-labor state, suggesting that repeated pregnancies in multiparous women may lead to chronic remodeling of the uterine wall. Repeated trauma increases susceptibility to infection, promotes connective tissue hyperplasia, and causes a loss of elasticity, all of which weaken the uterine wall. This further thinning impairs the uterus’s ability to withstand contractions, thereby increasing the risk of rupture. Placental implantation abnormalities, such as placenta accreta spectrum (PAS), also contribute to changes in the endometrium and myometrium. Uterine rupture in the second and third trimesters has been linked to PAS in 44% of cases (7). Additionally, autoimmune diseases (e.g., connective tissue diseases, systemic lupus erythematosus, antiphospholipid syndrome) contribute to uterine rupture through a variety of complex mechanisms. These include immune-mediated vascular damage, immune cell infiltration, hormonal interactions, and drug effects (e.g., corticosteroids). These factors lead to weakened myometrial tissue with insufficient repair capacity, thereby increasing the risk of uterine rupture (23–25).

Therefore, clinical risk assessment should not be limited to a history of uterine surgery. A more comprehensive review of obstetric history, particularly previous pregnancies and deliveries, is essential. It is crucial to maintain a high level of suspicion for uterine rupture in all pregnant women with acute abdominal pain, as early recognition is key to preventing adverse maternal and fetal outcomes.

### 4.4 Placenta accreta spectrum

In this study, three cases of uterine rupture were complicated by placenta accreta spectrum (PAS), with two involving scarred uteri and all occurring in second-trimester). This sequence–’cesarean scar – placenta accreta spectrum – spontaneous rupture in the second trimester’–represents a destructive and high-risk pathway. Mechanistically, a defect at the endometrial-myometrial interface impedes normal decidualization in the scarred region, leading to abnormal deep placental anchoring and trophoblast invasion (26). In the case of a scarred uterus, this defect further elevates the risk of PAS. The myometrial infiltration or absence at the implantation site renders this area the weakest part of the uterus, making it highly susceptible to rupture under the physiological tension generated by rapid uterine expansion during the second trimester.

Additionally, one PAS case in this study had a history of a previous vaginal delivery. This finding suggests that, although a cesarean scar is a well-established risk factor for PAS, prior vaginal delivery may also contribute to the development of PAS. This highlights the complexity and variability of risk factors for uterine rupture. These results emphasize the need for a more comprehensive and multifactorial approach to risk stratification in PAS patients. Placenta previa is one of the most significant independent risk factors for PAS. Moreover, direct damage to the endometrium from invasive gynecological surgeries, such as myomectomy, curettage, manual removal of the placenta, abortion, or other invasive uterine cavity procedures, further increases the risk of PAS. The greater the frequency of such procedures causing direct injury to the endometrium, the higher the likelihood of PAS occurrence (27). Non-surgical causes of endometrial damage, such as in vitro fertilization-embryo transfer (IVF-ET) (28) and uterine artery embolization, can also result in microtrauma to the endometrium, contributing to PAS development (29). Interestingly, PAS can also occur in women without a history of uterine surgery, including those with uterine anomalies, adenomyosis, or submucosal fibroids (30). Furthermore, advanced maternal age has been significantly associated with an increased risk of PAS (31). Several meta-analyses have demonstrated that factors such as multiple pregnancies, higher parity, and maternal obesity increase the risk of PAS by 1.5 to 2 times, further underscoring the multifaceted nature of PAS risk factors (32, 33).

Therefore, early prenatal diagnosis serves as the first line of defense in preventing uterine rupture. For women with a history of cesarean delivery, systematic prenatal ultrasound evaluations in early and mid-pregnancy are crucial for identifying this risk. Pagani et al. demonstrated that ultrasound signs such as “placental lacunae,” “myometrial thinning or disruption (<1 mm),” and “interruption of the bladder line” have high predictive value for diagnosing the depth of placenta accreta (34). These ultrasound findings should become routine assessments for women with a history of cesarean section. Early recognition of PAS facilitates risk stratification, the development of individualized prenatal monitoring plans, and delivery planning, potentially preventing catastrophic complications such as spontaneous rupture.

Clinical experience is essential to improving early diagnosis rates. Pregnant women suspected of PAS should be referred to tertiary care centers as soon as possible to improve pregnancy outcomes (35). A key reflection from this study is that three PAS cases were poorly documented in our hospital, and detailed imaging data prior to referral were unavailable. These cases may have lacked standardized ultrasound evaluations before transfer, missing the opportunity for early detection. Antenatal recognition of PAS is important because it allows for planned delivery and multidisciplinary management. However, the retrospective nature of this study and the limited availability of pre-referral imaging data make it difficult to determine whether PAS could have been reliably identified before rupture. If PAS had been diagnosed earlier, it would have facilitated a more informed assessment of the risks and benefits of continuing the pregnancy, allowing patients to make an informed decision, potentially preventing poor maternal and fetal outcomes.

### 4.5 Perinatal outcomes

In this study, the perinatal mortality rate was as high as 85.7% (6/7). Among these, four cases of uterine rupture occurred in the second trimester (<28 weeks), during which the fetus lacks the ability to survive outside the womb. The combination of acute placental abruption and maternal circulatory collapse due to uterine rupture led to catastrophic fetal outcomes. The remaining three cases, which occurred in the third trimester (1 survivor, 2 deaths), highlighted the critical importance of the “time window”–timely intervention is essential for improving fetal outcomes. Both the American College of Obstetricians and Gynecologists (ACOG) and the National Institute for Health and Care Excellence (NICE) recommend a decision-to-delivery interval (DDI) of 30 min or less, although these recommendations are largely based on expert consensus rather than high-level evidence (36, 37). While this time frame cannot serve as an absolute standard for all emergency cesarean sections, actively reducing the DDI has been shown to improve neonatal outcomes (38). The only surviving newborn in this study benefited from relatively prompt intervention. Therefore, for suspected cases of uterine rupture in the third trimester, an emergency response process aimed at saving both maternal and fetal lives must be initiated immediately, with the goal of minimizing the time from diagnosis to delivery.

### 4.6 Management and surgical approach

In this study, hysterectomy was performed in 4 cases vs. uterine repair in 3 cases, with the surgical approach determined by several clinical factors. Hysterectomy is indicated for extensive uterine rupture, particularly when major blood vessels are involved and bleeding cannot be controlled. It is also necessary in cases of persistent hemorrhage or when the mother’s condition remains unstable despite other hemorrhage control measures. Hysterectomy is required if the fetus is nonviable or maternal hemorrhage threatens survival. In contrast, uterine repair is preferred for localized ruptures, especially when bleeding is manageable and the rupture occurs in the lower uterine segment. Uterine repair is also suitable when the mother’s vital signs are stable, and no major blood vessels or critical structures are involved. When the fetus is still viable, uterine repair can help preserve both maternal and fetal health. Additionally, the choice of surgery is influenced by fertility considerations. For patients with no desire for future pregnancies or poor reproductive prognosis, hysterectomy is typically chosen, whereas uterine repair is favored in younger patients when the rupture is repairable to preserve fertility. Ultimately, the surgical decision is based on a comprehensive evaluation of maternal stability, fetal viability, and fertility preservation.

### 4.7 Limitations of the study

The conclusions of this study should be interpreted within the context of several important limitations. First, as a single-center retrospective case series with a small sample size (*n* = 7), the findings are limited in statistical power and generalizability. Second, all cases were referred as critically ill patients from other hospitals, introducing significant selection bias. This likely overestimates the severity of uterine rupture and perinatal mortality, while milder or more localized ruptures may be underrepresented. The focus on critically ill cases may reflect only the most severe presentations of the disease, which could skew the clinical features and outcomes. In clinical practice, uterine rupture can range from mild symptoms to life-threatening complications, but the referral process tends to select patients with more severe presentations, such as fetal distress, massive hemorrhage, or multi-organ failure. This selection bias inflates the perceived severity and perinatal mortality rates. Conversely, milder cases, which may present with less acute symptoms and be managed conservatively, are less likely to be referred to specialized centers and are thus underrepresented in this study. Consequently, the findings may not fully capture the spectrum of uterine rupture in the second and third trimesters.

Additionally, as a retrospective study, clinical details may have been missing or inconsistent, which further limits the accuracy of the findings. Furthermore, this study did not include patient perspectives, such as their experiences, treatment decisions, or long-term outcomes. The lack of patient-reported data limits the understanding of the psychological and emotional impact of uterine rupture on affected individuals and their post-intervention quality of life. These limitations suggest that the primary value of this study lies in generating hypotheses and providing detailed clinical descriptions, rather than confirming universal conclusions.

## Data Availability

The raw data supporting the conclusions of this article will be made available by the authors, without undue reservation.
